# The role of androgen and its related signals in PCOS

**DOI:** 10.1111/jcmm.16205

**Published:** 2020-12-23

**Authors:** Wenting Ye, Tingting Xie, Yali Song, Lili Zhou

**Affiliations:** ^1^ Division of Nephrology State Key Laboratory of Organ Failure Research National Clinical Research Center of Kidney Disease Nanfang Hospital Southern Medical University Guangzhou China; ^2^ Center for Reproductive Medicine Department of Obstetrics and Gynecology Nanfang Hospital Southern Medical University Guangzhou China; ^3^ Bioland Laboratory (Guangzhou Regenerative Medicine and Health Guangdong Laboratory) Guangzhou China

**Keywords:** androgen, cell signal, complication, hyperandrogenism, PCOS

## Abstract

Polycystic ovary syndrome (PCOS) is the most common endocrine disorder in women at reproductive age. However, the underlying pathogenic mechanisms have not been completely understood. Hyperandrogenism is an important clinic feature in patients with PCOS, suggesting its pathologic role in the development and progression of PCOS. However, the actual role of androgen and the related signals in PCOS and PCOS‐related complications have not yet been clarified. In this review, we surveyed the origin and effects of androgen on PCOS and the related complications, highlighted the cellular signals affecting androgen synthesis and summarized the pathological processes caused by hyperandrogenism. Our review well reveals the important mechanisms referring the pathogenesis of PCOS and provides important clues to the clinic strategies in patients with PCOS.

## 1 INTRODUCTION

Nowadays, that 5%‐20% of reproductive‐age women are suffering from PCOS. However, PCOS is still an intractable problem in medical society.

Hyperandrogenism is an important criterion for diagnosis to PCOS. In patients with PCOS, incidence rate of hyperandrogenism is as high as 60%‐80%. Androgen hyperactivation leads to ovulation disorder, menstrual disorder, hairy and acne, suggesting that hyperandrogenism is not only a clinical characteristic of PCOS, but also an important risk factor. The current anti‐androgen therapies in clinical settings have not achieved satisfactory effect, which lies in the complicated mechanisms of androgen production and its wide‐ranging effects. In this review, we comprehensively summarized the role of androgen and its related signals in PCOS and provided important clues to the clinical strategies in patients with PCOS.

## 2 ANDROGEN PHYSIOLOGY IN WOMEN

### 2.1 Physiological sources and classifications of androgen in women

**FIGURE 1:**
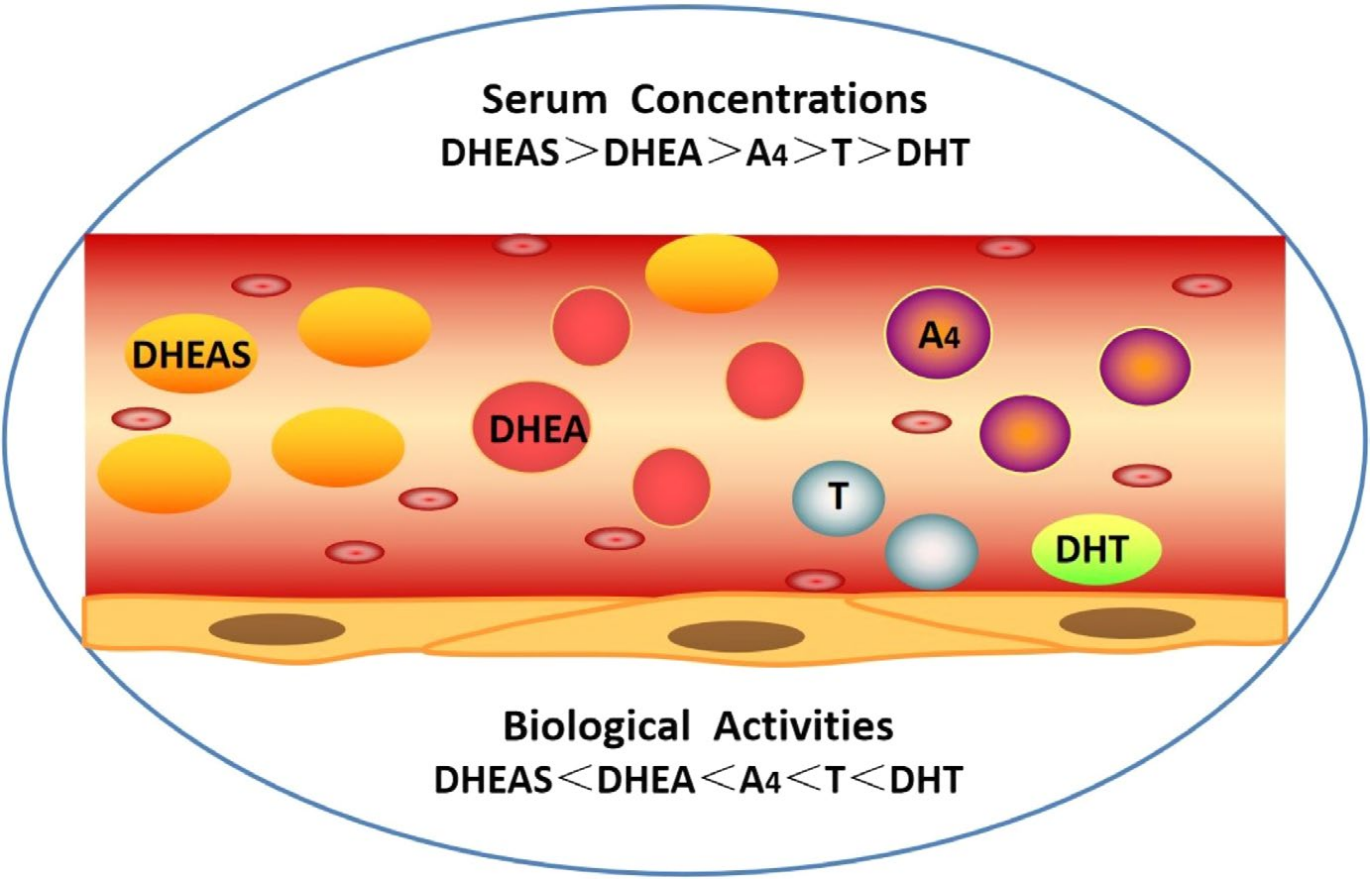
Serum concentrations and biological activities of different kinds of androgen. There are five types of androgen in women: dehydroepiandrosterone sulphate (DHEAS), dehydroepiandrosterone (DHEA), androstenedione (A_4_), testosterone (T) and dihydrotestosterone (DHT). Serum androgen concentration in women is DHEAS > DHEA > A_4_ > T>DHT. Androgen biological activities: DHEAS < DHEA < A_4_ < T<DHT

In women, androgen is mostly originated from cholesterol through the action of luteinizing hormone (LH) from theca cells in ovary and adrenocorticotropic hormone from adrenal zona reticularis. A small portion of androgen could still be produced by peripheral tissues.^1^ There are five types of androgens in women, including dehydroepiandrosterone sulphate (DHEAS), dehydroepiandrosterone (DHEA), androstenedione (A_4_), testosterone (T) and dihydrotestosterone (DHT).^2^ DHEAS is only produced by the adrenal zona reticularis. DHEA is secreted by adrenal zona reticularis (50%), ovaries (20%) and conversion from DHEAS in circulation (30%).^2^ A_4_ is synthesized equally by adrenal gland and ovary.^2^ As a bioactive form of androgen, T is a secreted product equally from the adrenal zona fasciculata and ovary. DHT is mainly transformed from T by 5α‐reductase (5αRD) in the peripheral tissue such as liver, adipose tissue and the pilosebaceous unit.^2^ A small amount of DHT is also synthesized by the adrenal zona fasciculata. Their serum concentrations are in descending order, whereas the biological activities are opposite (Figure 1). DHEAS, DHEA and A_4_ are the major androgen precursors, whereas testosterone and DHT are potent androgens that induce biological effects through directly binding to androgen receptor (AR). All androgens are metabolized by the liver, and their metabolic wastes are excreted through urine.

### 2.2 Androgen synthesis in ovary

Androgen is biosynthesized from cholesterol by the theca cells in ovary.^1^ After stimulated by LH, cholesterol is transferred into mitochondria via the activation of steroidogenic acute regulatory protein (StAR) and then converts to pregnenolone through the enzyme P450 side chain cleavage (P450scc, CYP11A1). Then, pregnenolone transfers to smooth endoplasmic reticulum from mitochondria and would be transformed into 17‐hydroxypregnenolone by the 17‐hydroxylase subunit of cytochrome P450c17 (CYP17A1) (an enzyme containing two active sites of 17‐hydroxylase and 17, 20‐lyase).^3^ DHEA is metabolized from 17‐hydroxypregnenolone by 17, 20‐lyase subunit of CYP17A1. DHEA could convert into A_4_ by ∆5‐isomerase‐3β‐hydroxysteroid dehydrogenase type 2 (3βHSD2). 3βHSD2 can also transform pregnenolone and 17‐hydroxypregnenolone into progesterone and 17‐hydroxyprogesterone, respectively. The transforming pathway of progesterone to A_4_ follows the same pathway as pregnenolone. Then, A_4_ can be converted to testosterone and estrone by 17β‐hydroxysteroid dehydrogenase type 5 (17βHSD5, HSD17B5) in the theca cells and cytochrome P450aro (CYP19A1) in the granulosa cells. Subsequently, in granulosa cells, testosterone is transformed to DHT and estradiol by 5α‐reductase and CYP19A1, respectively.^3^ Estrone can finally be converted to estradiol by 17β‐hydroxysteroid dehydrogenase type 1 (17βHSD1, HSD17B1).

### 2.3 Physiological effects of androgen in women

Among all androgens, testosterone and DHT are more active than others. The activity of DHT is 3 fold higher than testosterone.^4^ About 66% of testosterone is bound to sex hormone–binding globulin, and 33% is bound to albumin, whereas merely 1% is free.^5^ It is noteworthy that only free testosterone exhibits biological activity, whereas the protein‐bound testosterone is inactive.^5^ Similarly, only free DHT is bioactive. Hence, the circulating concentrations of free testosterone and DHT are responsible for biological effects. The biological effects of androgen rely on their binding to AR in target cells.

Testosterone could be transformed into estradiol by aromatase. Testosterone and estradiol coordinate to maintain the balance of female reproductive endocrine system. In the peripheral organs, androgen helps to increase muscle mass, bone growth, and calcium deposition.^6^ The appearance of pubic hair and axillary hair, an indication of puberty, also relies on the secretion of androgens from adrenal gland, known as the adrenarche.^7^ However, excessive androgen can cause follicular dysplasia, which may impair ovulation and would result in menstrual disorders. In addition, androgen is an important hormone for maintaining the female's sexual desire. Therefore, it is important to maintain the balance between androgen synthesis and secretion.

## 3 ANDROGEN SYNTHESIS AND THE ROLE OF ANDROGEN IN PCOS

PCOS is the most known endocrine disorder in female at reproductive age. Hyperandrogenism has been recognized as a contributor to aggravate the reproductive symptoms and the development of metabolic syndrome in PCOS. It is believed that the excessive androgen is primarily from ovary and the adrenal gland. Although adrenal hyperandrogenism affects 20%–30% of PCOS patients,^1^ it does not exert any effect on metabolic disorder in PCOS patients.^8^ However, excessive androgen from ovaries is considered to be the most important inducer of PCOS.^1^ Hence, in the following sections, we mainly focused on androgen synthesis in the ovaries of PCOS patients and summarized its cellular activities.

### 3.1 Androgen synthesis in PCOS

#### 3.1.1 The GPCR/cAMP/PKA pathway

G protein‐coupled receptor (GPCR) is the most widely expressed receptor in the cell membrane. Steroidogenesis is mainly related to two types of GPCRs. One is melanocortin 2 receptor (MC2R), the specific receptor for adrenocorticotropic hormone (ACTH).^9^ ACTH is well‐known for its initial ability to induce steroidogenesis in adrenal gland. Through binding to MC2R, ACTH promotes the production of adenylate cyclase (cAMP) and then activates protein kinase A (PKA) to further induce androgen synthesis. The other receptors include FSH receptor (FSHR) and LH receptor (LHR) responsive for FSH and LH, respectively. FSHR and LHR also mediate steroid biosynthesis in the gonads through cAMP/PKC pathway.^10^ The cAMP/PKA pathway is widely known as the major signal pathway in regulating steroidogenesis, as it is a master pathway to induce multiple downstream signals.

#### 3.1.2 The PI3K/AKT pathway

The PI3K (phosphatidylinositol 3‐kinase)/AKT (protein kinase B) pathway is a major non‐gonadotropic signalling pathway. PI3K/AKT pathway overactivation is intimately correlated with female reproductive diseases, especially PCOS. In addition, PI3K/AKT signalling pathway is closely associated with insulin resistance (IR) and adipocyte differentiation, which highly influences the disease progression of PCOS.^11^ These effects are highly related to androgen synthesis induced by PI3K/AKT pathway.

Overactivated PI3K/AKT signals in theca cells could lead to androgen excess and ovarian dysfunction. CYP17A1 is a key enzyme for androgen synthesis and highly expressed in PCOS.^12^ In theca cells, it is stimulated by LH through activation of PI3K/AKT pathway.^13^ Furthermore, CYP17A1 and 17α‐hydroxylase are also involved in insulin‐induced testosterone biosynthesis in theca cells,^14^ through IRS (insulin receptor substrate)/PI3K pathway and the coactivation of the cAMP signalling.^15^ In addition, StAR (steroidogenic acute regulatory protein) and CYP11A1 are also highly expressed in PCOS^16^ and positively correlated to the activation of PI3K/AKT signalling.^17^ Notably, metformin, a common therapy for PCOS‐related complications, attenuates androgen production in theca cells by inhibiting PI3K/Akt signalling.^18^

#### 3.1.3 The MAPK signal pathway

Mitogen‐activated protein kinase (MAPK) pathways are known to be related to many diseases. There are three pathways involved: (a) mitogen‐activated protein kinase 1/2 (MEK1/2)/extracellular signal‐regulated kinase 1/2 (ERK1/2), (b) p38MAPKs and (c) c‐Jun N‐terminal kinases/stress‐activated protein kinases (JNK/SAPKs). Among them, ERK1/2 is more related with the pathophysiology of PCOS, as it is highly involved in androgen and oestrogen metabolism.

Reports have shown that in PCOS‐affected granulosa cells (GCs), MAPK kinase kinase 4 (MAP3K4) and phospho (p)‐ERK1/2 were down‐regulated and this would influence GCs' function,^19^ suggesting its high correlation with excessive production of androgen. A study has shown that inhibition of MAPK signalling in GCs prompts the expression of CYP17 (cytochrome P45017A1).^20^ Another study has shown that the expression of StAR in GCs in patients with PCOS remains lower and is related to the inhibition on ERK activity.^21^ However, the article is controversial, as more and more scholars have found the increase of StAR in PCOS.^16^ If not, at least, the level of StAR is close to normal.^16^ Other studies have also shown the different observations. MAPK cascade could also down‐regulate StAR and CYP17A1 expression to inhibit gonadotropins‐induced androgen synthesis.^22^, ^23^ In addition, the crosstalk between cAMP and MEK/ERK could influence androgen synthesis, mainly through regulating the expression of 3βHSD2 and StAR.^24^ Nevertheless, further studies are needed to clarify the underlying mechanisms.

#### 3.1.4 The Wnt signal pathway

Wnt signalling is an evolutionarily conserved pathway involving in cell growth, proliferation, migration and apoptosis. Recently, it has been demonstrated that Wnt signal plays an important role in PCOS, possibly through its inducing effect on androgen synthesis.

Wnt4 could affect female sexual development and ovarian function in a paracrine manner. Wnt4 mutation enhances androgen synthesis, probably by increasing the expression of CYP11A1, CYP17, 17βHSD1 and StAR.^25^ However, whether Wnt4 plays a role in PCOS needs to be further studied, as a study shows that no causative mutation in Wnt4 has the correlation with PCOS,^26^ whereas another study shows a high expression of Wnt4 in patients with PCOS.^27^ The high level of Wnt4 is accompanied by cell apoptosis in GCs.^27^ These results also suggest the intimate correlations between Wnt4 and androgen excess.

More studies are needed to identify the legitimate role of Wnt signalling in PCOS and androgen hyperactivity as there are 19 Wnt members in mammals and some studies also show hyperandrogenemia occurs with attenuated Wnt signalling.^28^

**FIGURE 2:**
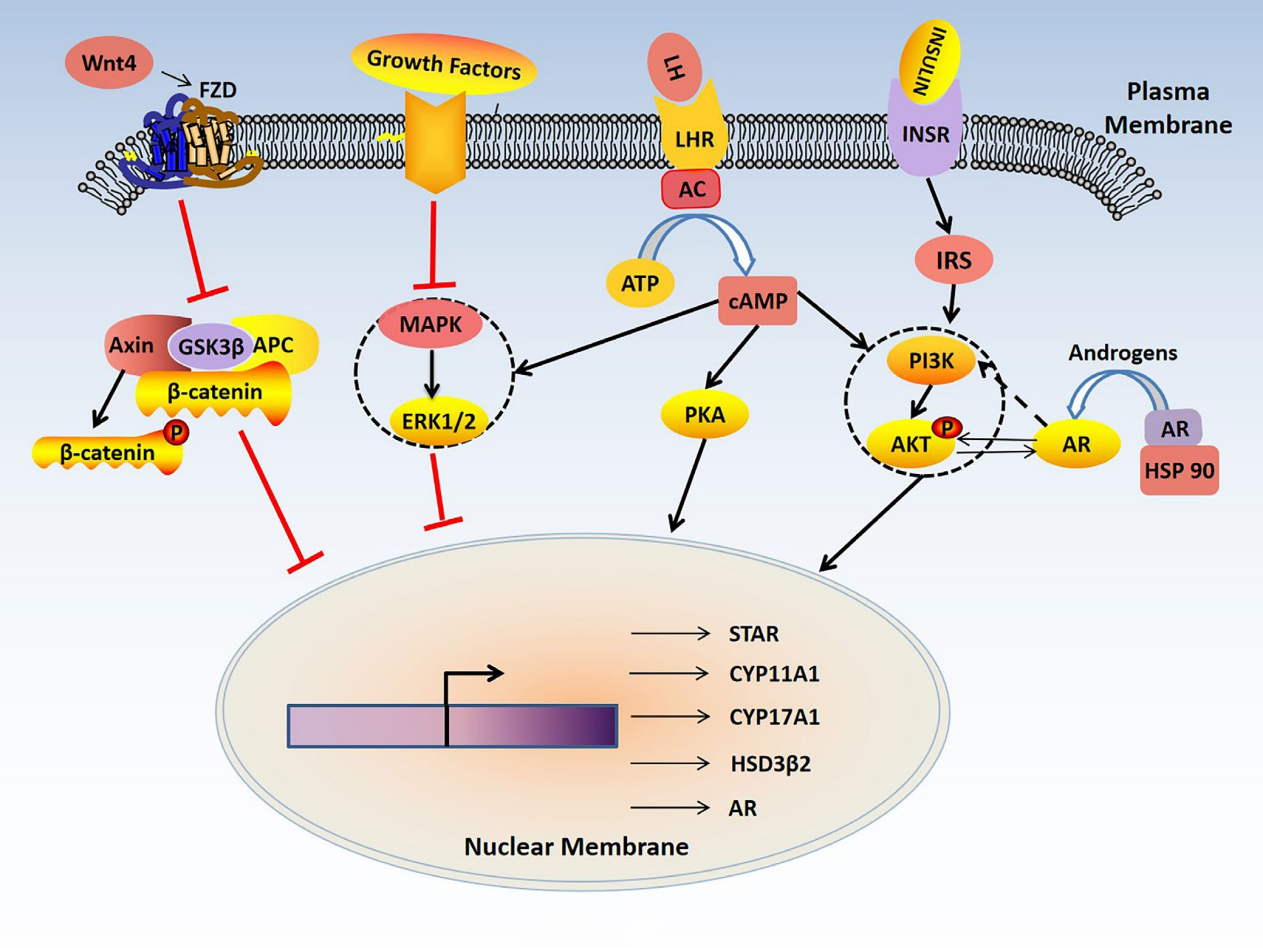
The signal pathways related with androgen synthesis in polycystic ovary syndrome (PCOS). The GPCR (G protein‐coupled receptor)/cAMP (adenylate cyclase)/PKA (protein kinase A) pathway is a classic signal pathway in regulating androgen synthesis in theca cells. Activation of PI3K (phosphatidylinositol 3‐kinase)/AKT (protein kinase B) signal and inhibition of MAPK (mitogen‐activated protein kinase) and Wnt signals are involved in androgen synthesis. In addition, cAMP can cross‐talk with PI3K/AKT and MAPK signals. Interaction between androgen receptor (AR) and PI3K/AKT signaling also affects androgen synthesis

As summarized above, the related signal pathways in androgen production are shown in Figure 2. They form a complex network in a mode of crosstalk to cooperatively affect androgen biosynthesis.

### 3.2 The role of androgen in PCOS

The abnormality of the menstrual cycle is also one of the critical characteristics in patients with PCOS. Accompanied by the prolongation of the menstrual cycle, anovulation becomes more frequent, which further leads to amenorrhoea, endometrial hyperproliferation and even carcinogenesis. PCO is another vital feature in PCOS women, defined as the presentations of at least 12 antral follicles (AFC) with a diameter from 2 to 9 mm in the whole ovary and/or an ovarian volume over 10mL. Excessive AFC could lead to secretion of large amounts of oestrogen, which inhibits the secretion of follicle‐stimulating hormone (FSH) via a negative feedback of the gonadal axis and leads to anovulation. Therefore, the length of the menstrual cycle and the number of AFC in ovaries are the two main indicators to estimate the disease severity of PCOS. The serum level of testosterone is positively correlated with the length of the menstrual cycle and the numbers of AFC,^29^ suggesting that hyperandrogenism is a promoter to the development and progression of PCOS. In addition, excess androgen can damage GCs and change the microenvironment of follicles, resulting in follicular atresia in PCOS.^30^ The overview of this is given below.

### 3.3 Effects of androgen on cell activities in PCOS

#### 3.3.1 Apoptosis

Apoptosis plays an important role in the physiological growth and development of follicles, whereas overactivated apoptosis affects the development of oocytes by transmitting apoptotic information from GCs to oocytes.

Excessive androgen can induce cell apoptosis through multiple signal pathways. The high expression of Klotho is involved in this pathway.^31^ Some epigenetic changes are also involved. The aberrant expression of microRNAs (miRNAs) is involved in androgen‐induced GCs apoptosis.^32^ Furthermore, the demethylation of programed cell death 4 (PDCD4) is also intimately correlated with the apoptosis in human GCs.^33^

Hyperandrogenism in PCOS might also directly induce apoptosis in oocytes. Heat shock protein 27 (HSP27), an anti‐apoptotic protein of HSP family, is significantly down‐regulated in oocytes of patients with PCOS.^34^ Lower expression of HSP27 could affect multiple signalling pathways in the ovaries and contribute to the abnormal oocyte development, a characteristic of PCOS.^34^ It might be speculated that androgens are involved in the changes in oocytes, as they participate in regulating gene expression in the oocytes.^35^ In addition, androgen promotes primordial follicle activation via the PI3K/AKT/FOXO3a (forkhead box O3) pathway,^36^ but it inhibits the expression of growth and differentiation factor 9 (GDF9) in oocytes and thus blocks the transition from primary to secondary follicles,^36^ which may ultimately lead to oocyte apoptosis.^37^

#### 3.3.2 Autophagy

Autophagy mediates follicle growth, atresia and differentiation. Abnormal autophagy can lead to cell death, which thus influences the development and quality of oocytes. Several studies have shown that androgen and AR may play an important role in activating autophagy.

A recent study has shown that autophagy is activated in PCOS, and the autophagy‐related network involving EGFR, ERBB2, FOXO1, MAPK1, NFKB1, IGF1, TP53 and MAPK9 is responsible for the activation.^38^ In addition, the accumulation of autophagosomes could lead to GC apoptosis. Excessive androgen could significantly induce autophagy‐related genes *ATG5, ATG7, BECLIN1* and the ratio of autophagy marker protein light chain 3B II/I (LC3 II/I) in patients with PCOS.^39^

However, whether autophagy is activated remains controversial. A study from Kobayashi et al identifies that the accumulation of p62 and ubiquitin, an aggregated protein and impaired autophagy markers, are presented in the theca cell layer in PCOS, indicating the impairment of autophagy.^40^ They propose that the dysregulation of late‐stage autophagy in theca cells induce androgen production and fibrosis via the reactive oxygen species (ROS)/p38 and JNK signalling pathway.^40^

#### 3.3.3 Mitochondrial dysfunction

Several studies reported that mitochondrial dysfunction is involved in the development PCOS,^41^ and androgen excess may lead to mitophagy and autophagy‐induced cell death.

Dynamin‐related protein 1 (Drp1), a cytoplasmic GTPase involved in mitochondrial fission, could be up‐regulated by DHT through the macroautophagy and apoptosis in PCOS‐affected GCs.^42^ Furthermore, mitochondrial dysfunction accompanied by abnormal glycolysis in patients with PCOS affects the switch of energy metabolism to glycolytic, resulting in GCs dysfunction and poor oocyte competence in PCOS.^43^ Another study shows that mitochondrial dysfunction in PCOS‐affected GCs can be improved by vitamin D3 through stimulating MAPK signalling.^44^

In PCOS mouse model, impaired inner mitochondrial membrane function, elevated ROS production and increased RNA transcript abundance are also found in oocytes.^45^ Furthermore, other studies have shown that oxidative stress and DNA methylation loci in oocytes could lead to mitochondrial dysfunction^46^ and may further contribute to androgen excess.^35^

#### 3.3.4 Endoplasmic reticulum stress

Endoplasmic reticulum (ER) stress is caused by a series of physiological and pathological conditions, which results in the activation of the unfolded protein response (UPR) cascades and affects diverse cellular functions.

Endoplasmic reticulum stress is activated in GCs. Androgen excess could induce the expression of *UPR* genes including transcription factor C/EBP homologous protein (CHOP) and death receptor 5 (DR5) in PCOS‐affected GCs, which results in ER stress and cell apoptosis.^47^ Furthermore, the accumulation of advanced glycation end products (AEGs) in GCs is caused by androgen‐triggered ER stress,^48^ which may contribute to the metabolic and reproductive consequences of PCOS.^49^ Resveratrol may be a possible treatment strategy for ER stress in PCOS.^50^

Endoplasmic reticulum stress in oocytes has not yet been widely studied in human and animals. Deterioration of oocyte quality is associated with ER stress in oocytes.^51^ However, oocyte incompetence in PCOS with androgen excess is widely accepted, it can be speculated that androgen‐induced ER stress in oocytes is a possible mechanism for poor developmental competence in PCOS‐affected oocytes. However, further studies are needed to confirm this.

**FIGURE 3:**
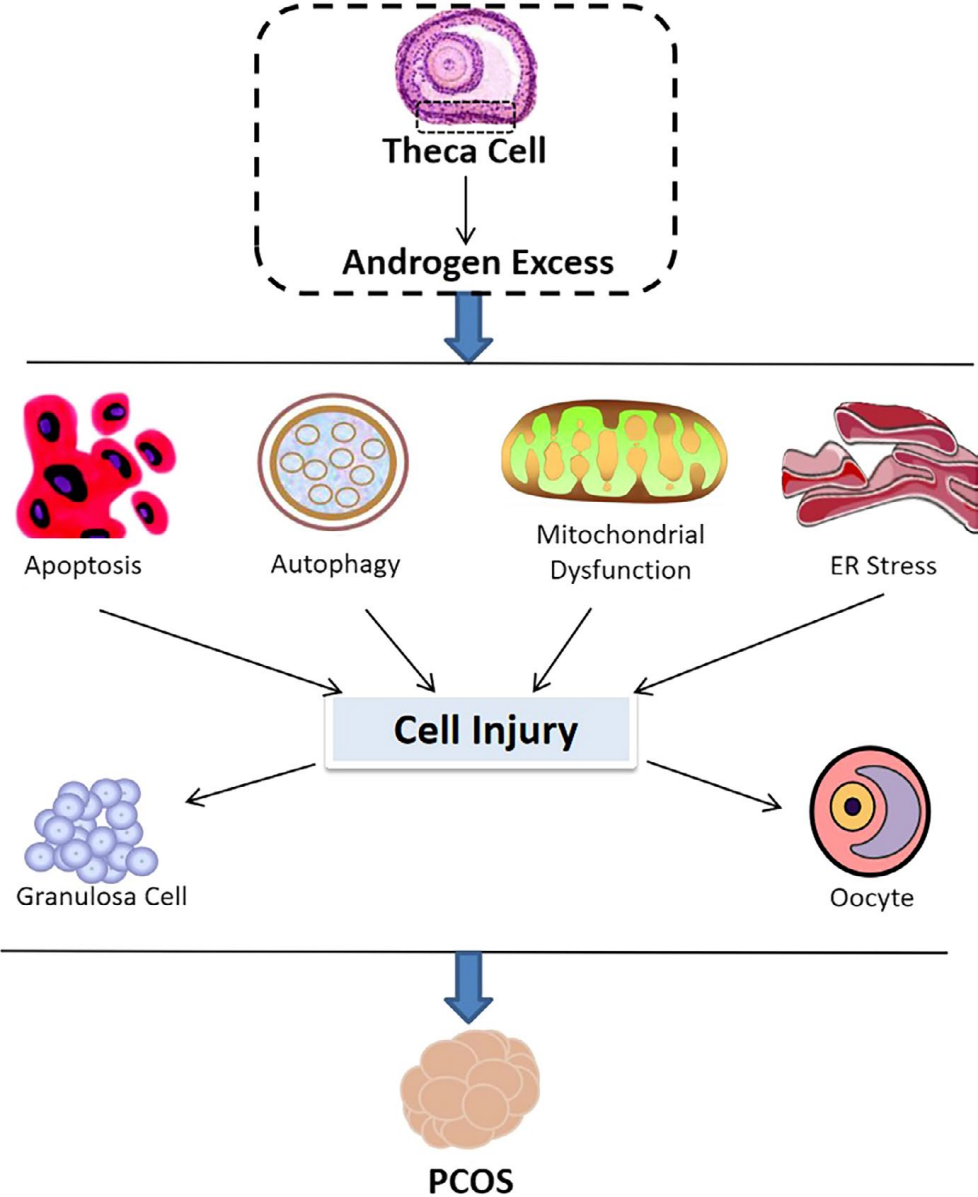
Effects of androgen on cell activities in PCOS. Excessive androgen stimulates and promotes polycystic ovary syndrome (PCOS) by inducing cellular activities such as apoptosis, autophagy, mitochondrial dysfunction and endoplasmic reticulum stress in granulosa cells and oocytes

A summary of studies mentioned above is shown in Figure 3. Although apoptosis induced by androgen excess is commonly recognized by researchers, autophagy, mitochondrial dysfunction and ER stress are also highly related to hyperandrogenism and should not be neglected.

## 4 THE ROLE OF ANDROGEN IN PCOS‐RELATED COMPLICATIONS

**FIGURE 4:**
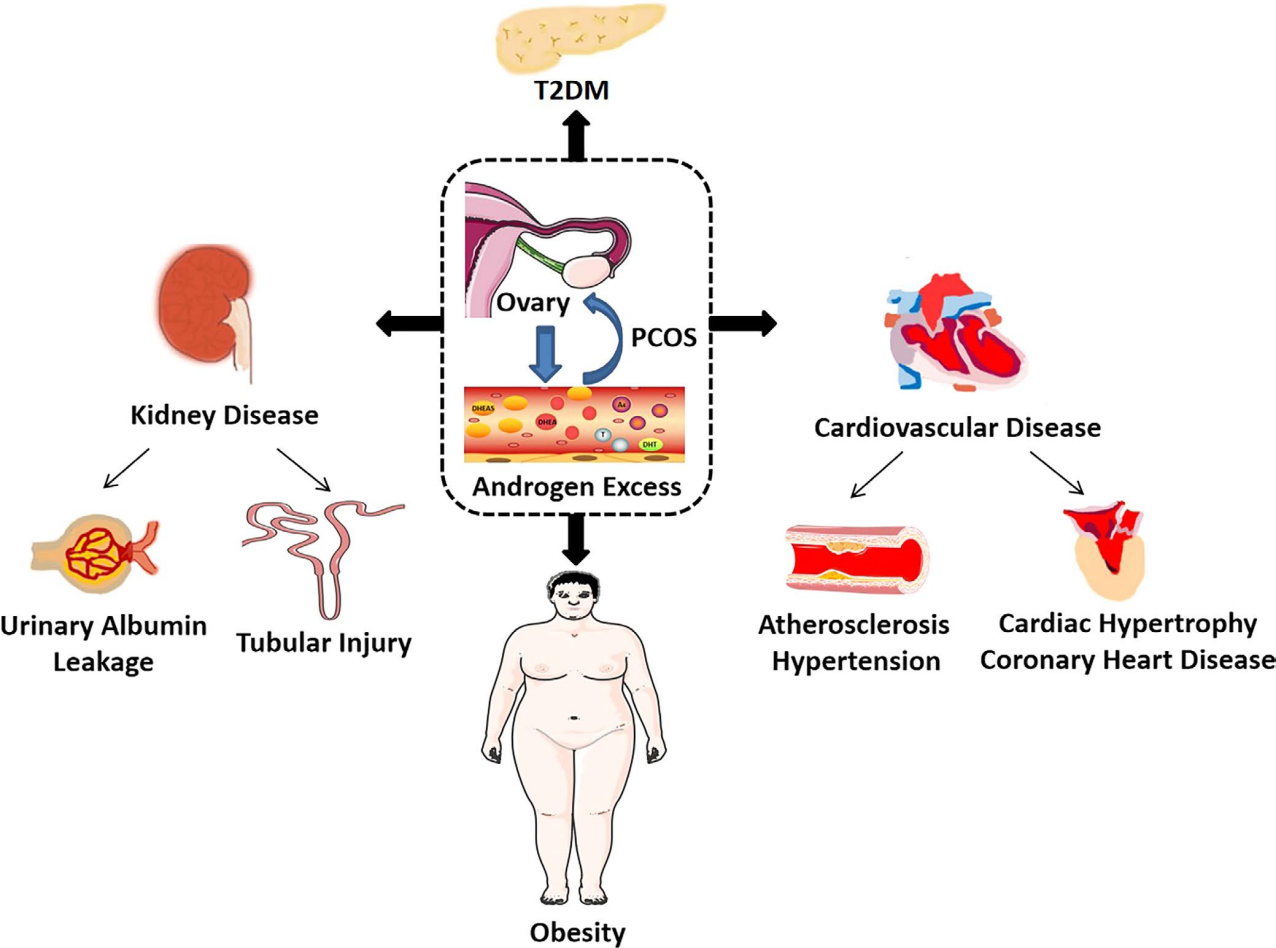
The role of androgen in PCOS and PCOS‐related complications. Excessive androgen not only leads to polycystic ovary syndrome (PCOS) in the ovaries but also promotes many metabolic disorders such as cardiovascular disease, type 2 diabetes mellitus, kidney disease and obesity

Polycystic ovary syndrome has an intimate link to many disorders such as metabolic syndrome. Although insulin resistance serves as an important risk factor for metabolic syndrome and other PCOS‐related diseases, hyperandrogenism could also be an independent risk factor for type 2 diabetes, obesity, cardiovascular diseases (CVD) and metabolic syndrome in female patients. We next summarized the role of hyperandrogenism in some typical complications of PCOS (Figure 4).

### 4.1 Obesity

Obesity is reported to be the most common feature in patients with PCOS (33%–88%). Obesity has a great impact on fertility and can lead to adverse consequences such as menstrual disorders, anovulation, infertility and abortion. Hence, it is of great significance to manage weight at an early stage of PCOS for improving the ability of childbearing and quality of life.

Hyperandrogenism plays a critical role in abdominal obesity in obese women during adolescence, adulthood and menopause.^52^ Although a study has reported the negative association between plasma androgen levels (A4, DHEA and DHEAS) and obesity,^53^ most of the existing literature show that the increase in body weight in women with PCOS may result from hyperandrogenic state.^54^

Notably, there would be gender difference about the effects of androgen. Androgen/AR signalling in males can stimulate the commitment of pluripotent mesenchymal stem cells into myogenic lineage but suppress the adipogenic lineage through down‐regulating the expression peroxisomal proliferator‐activated receptor γ 2 (PPARγ2) and CCAAT/enhancer binding protein α (C/EBPα).^55^ Knockdown of AR in male mice would develop the late onset visceral obesity and increase lipogenesis in adipose tissue and liver.^56^, ^57^

But the mechanism about how androgens affect fat cells in women is poorly understood. Other studies may give some inspirations. They indicate that excessive androgens could directly increase the proliferation in visceral preadipocytes through activating APOBEC3b (apolipoprotein B mRNA editing enzyme catalytic subunit 3B), CCNA2 (cyclin A2) and PRC1 (protein regulator of cytokinesis 1),^58^ and at the same time, accompanied with the overactivated CMKLR1 signalling which causes the accumulation of lipid droplets.^59^ In addition, androgen could also inhibit the differentiation of the subcutaneous mesenchymal stem cells by inhibiting bone morphogenic protein 4 (BMP4) signalling^60^ and by switching macrophages polarization into M2 through the G‐protein and Akt signalling pathways.^61^ Furthermore, reduction of catecholamine‐stimulated lipolysis induced by excessive androgen is also another potential mechanism.^62^ This accelerates the accumulation of ectopic fat, such as liver fat, thereby leading to visceral obesity.^63^ However, despite that the effects of androgen on fat cells show gender difference, the underlying mechanism still remains unknown. Further studies are needed to clarify it.

Additionally, androgen and adipose tissue could aggravate each other in a vicious cycle in that adipose tissue could act as a hormone supplier and modulator. Reports show that some steroidogenic enzymes are expressed in adipose tissue, for example, aromatase, 3βHSD, 3αHSD, 11βHSD, 17βHSD, 7α‐hydroxylase, 17α‐hydroxylase and 5αRD.^64^ In addition, local activation of androgen in adipose tissue may be greater in mediating fat mass expansion than the increased circulating form.^65^ Hence, excessive androgen plays a critical role in the pathogenesis of obesity, especially abdominal obesity in patients with PCOS.

### 4.2 T2DM

Hyperandrogenism is also a risk factor for T2DM. Several studies have indicated a high incidence of T2DM in all adolescent and pregnant patients with PCOS. Hyperinsulinemia and overactivated accumulation of fat cells in diabetic disease state can aggravate hyperandrogenism,^66^ and vice versa. Various studies have confirmed the pathogenic role of androgen in the onset of diabetes in women. Higher levels of androgen are linked to the impaired glucose tolerance (IGT) and IR in women.^67^ In addition, glycosylated haemoglobin (HbA1c) and serum insulin levels increase concomitantly with free androgen index (FAI), whereas insulin sensitivity and β‐cell function decrease, suggesting the intimate relationship between T2DM and PCOS.^67^

It has been elaborated that in males with androgen deficiency, testosterone/AR regulates the key enzymes and proteins for glucose metabolism (eg PEPCK (phosphoenolpyruvate carboxykinase), glycolytic enzymes, and GLUT4 (glucose transporter type 4), PPARγ (peroxisome proliferator‐activated receptor γ) and LXRα (liver X receptor α).^68^, ^69^ However, the effects of androgen in female glucose metabolism are different from males. Androgen levels in women are positively correlated to the increase in PPARγ.^70^ Similarly, excessive DHT in female mice could induce insulin hyper‐secretion in an AR‐dependent cAMP‐ and mTOR‐dependent pathway.^71^ Furthermore, testosterone could induce endoplasmic reticulum stress and ultimately lead to apoptosis in β‐cell through eIF2α/CHOP cascades.^72^ Mitochondrial dysfunction, such as increased mitochondrial aspiration and oxygen consumption, is also involved in androgen‐induced β cell dysfunction.^73^, ^74^ Taken together, the aforementioned studies indicate excessive androgen in women could directly impair β‐cell function, further leading to glucose metabolism disorder.

However, the mechanism of gender differences in androgen‐induced metabolic disease is still poorly understood. And the effects of androgen on T2DM in PCOS also remain to be clarified.

### 4.3 Hypertension and atherosclerosis

Hypertension and atherosclerosis are increasingly prevalent in patients with PCOS. The excessive androgen could independently aggravate the development of hypertension and atherosclerosis in patients with PCOS.^75^

Roles of androgen in blood pressure also present gender difference. Reports show that the increased blood pressure is positively correlated to males with androgen deficiency and women with androgen excess.^76^ Among the various types of androgens, testosterone is regarded to be the predominant endogenous androgen in the bloodstream responsible for the hypertension.^77^ Studies have shown that androgen can modulate vascular endothelial growth factor (VEGF), matrix metalloproteinase (MMP)‐9 and 20‐hydroxyeicosatetraenoic acid (20‐HETE), accounting for the pathophysiology of hypertension.^78^, ^79^ And inhibition of IκB kinase (IKK) relieves the androgen‐20‐HETE‐mediated increase in blood pressure.^79^ In addition, another mechanism is renin‐angiotensin system (RAS) activation in kidneys and adipose tissue, which is mediated by the up‐regulation of androgen and its receptors in these organs.^80^ Furthermore, testosterone could induce vascular smooth muscle cells (VSMCs) migration via NADPH oxidase–derived ROS production and c‐Src–dependent pathways,^81^ and this is closely related to hypertension.^81^

Some studies reported that excessive androgen could increase the carotid intima‐media thickness and calcification in coronary and aortic artery in patients with PCOS,^82^, ^83^ which may reflect atherosclerosis induced by androgen excess.^84^ Other reports also show that androgen could induce endothelial dysfunction.^85^ As an early marker of CVD, endothelial dysfunction has been identified at an early stage of PCOS. This is independent of obesity ^86^ and leads to vasodilatation impairment and the release of endothelin‐1 (ET‐1) (an important marker of endothelial injury), which further aggravates endothelial injury.^87^ This may explain androgen‐induced atherosclerosis in PCOS, as impaired endothelial function is also one of the early processes in the pathophysiology of atherosclerosis.

### 4.4 Cardiac hypertrophy and coronary heart disease

Cardiac diseases are characterized with coronary heart disease, myocardial infarction, angina pectoris, heart failure and stroke, all of which would lead to a high mortality in women. A series of reports have shown a high risk of cardiac diseases in patients with PCOS, such as cardiac hypertrophy and coronary heart disease^88^ and hyperandrogenic phenotypes increase the risk of them.^89^

Studies show that cardiac hypertrophy, especially left ventricular hypertrophy, is associated with hyperandrogenism in PCOS. Prenatal exposure to androgen would programme long‐lasting heart remodelling in female mouse offspring^90^ and highlight the potential risk of cardiac dysfunction in the female offspring.^90^ The underlying mechanisms of cardiac hypertrophy in PCOS with hyperandrogenism are summarized as following. On one hand, excessive androgen could induce the activation of cardiac insulin signalling and thereby result in the activation of PI3K/AKT/mTORC1, which indirectly leads to cardiac hypertrophy.^91^ On the other hand, the intimate coordinated work of androgen and Ca^2+^‐dependent signalling indicates another potential mechanism. Duran J et al reported that AR signalling cooperates with Ca^2+^/calmodulin‐dependent protein kinase II (CaMKII) to activate myocyte‐enhancer factor 2 (MEF2), thereby inducing cardiac hypertrophy.^92^ Another study shows that testosterone participates in the activation of calcineurin/nuclear factor of activated T cells (CaN/NFAT) and the inhibition of GSK‐3β, which contributes to the development of cardiac myocyte hypertrophy.^93^

Coronary heart disease is commonly reported in PCOS,^94^ but there is no research on the direct role of hyperandrogenism in PCOS. However, in post‐menopausal women, a higher testosterone/estradiol ratio triggers a high risk for developing incident coronary heart disease and heart failure events,^95^ and this may result from testosterone‐induced apoptosis in cardiomyocytes.^96^ As an aggravating factor of CVD,^75^ hyperandrogenism might also increase the incidence of coronary heart disease in PCOS. Further studies are needed to clarify it.

Although progress has been made in understanding the relationship between CVD and PCOS, further studies are needed to elucidate the underlying mechanism. This is because some reports have pointed out that there are multiple biases in the current PCOS cohorts' studies^97^ and could overestimate the correlation.

### 4.5 Kidney diseases

Chronic kidney disease (CKD) is manifested as a decrease in glomerular filtration rate (GFR; GFR < 60 mL/min) over 3 months and proteinuria. Due to the high correlation between CKD and metabolic disorders, there may be a close correlation between PCOS and kidney diseases. Studies have revealed that there is premicroalbuminuria and an increase of cystatin C (a biomarker for renal function) in PCOS women.^98^, ^99^

Considering that there are abundant androgen receptors in renal cells, such as mesangial cells and proximal tubular cells, excessive androgen could be a causal risk factor for kidney diseases. Reports show that androgen/AR imposes the susceptibility to severe infections in the upper‐tract urinary tract and a high rate of urinary citrate and sodium excretion in women.^100^, ^101^ Furthermore, we previously found a significantly positive correlation between serum testosterone in patients with PCOS with renal tubular cell injury,^102^ and the follicular fluid collected from patients with PCOS could induce fibrotic lesions in cultured renal proximal tubular cells.^102^ Similarly, other reports have shown that there is an increase in urinary albumin excretion, glomerular injuries and interstitial fibrosis, and significant reduction in GFR in some female (HAF) rats with hyperandrogenemia.^103^

However, the specific mechanism is not mentioned in the aforementioned studies. It is reported that testosterone could induce the apoptosis in renal tubular epithelial cells and necrosis by activating the HIF‐1α/BNIP3 (Hypoxia inducible factor 1α/Bcl‐2 interacting protein 3) pathway.^104^ Another study has shown that androgen/AR and Fgf10/Fgfr2 signalling participate in renal fibrosis.^105^ In addition, evidence shows that decreased androgens protects against renal injury by reducing T cell infiltration and enhancing anti‐inflammatory cytokine production.^106^ Studies also show that prenatal testosterone induces proteinuria in adulthood,^107^ which may explain the results of premicroalbuminuria in patients with PCOS. The detailed mechanisms involving in PCOS‐related kidney injury are still in limitation, and more studies are needed for further exploration.

**FIGURE 5:**
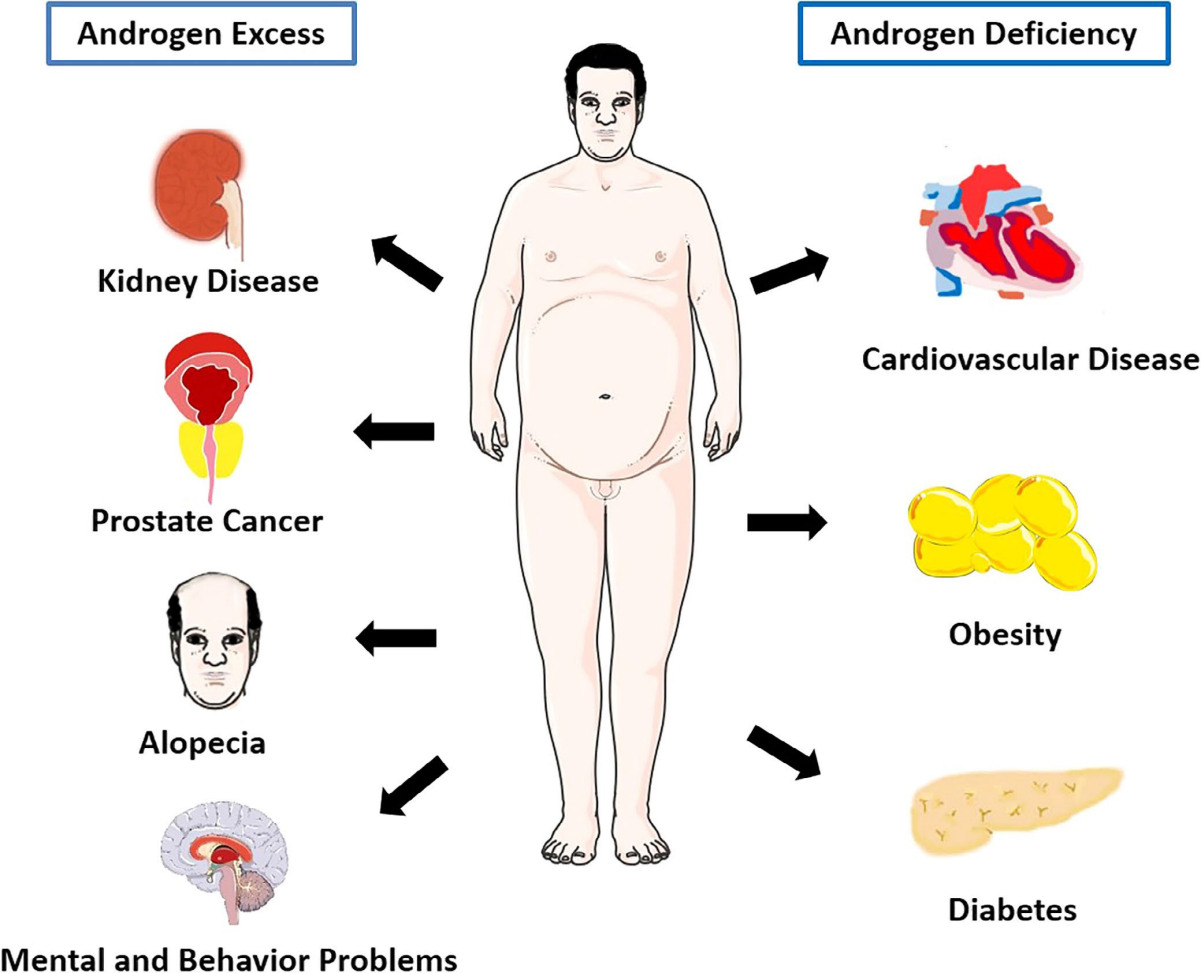
Androgen dysfunction leads to various diseases in men. Androgen homeostasis in men plays a very important role in maintaining body health. Excessive androgen in men could lead to kidney disease, prostate cancer, alopecia, as well as mental and behaviour problems, whereas androgen deficiency in men could cause cardiovascular disease, type 2 diabetes and obesity

Although the aforementioned studies have implicated the possible molecular mechanisms of hyperandrogenism in PCOS‐related complications, its true nature remains to be explored. Notably, there would be gender difference about the effects of androgen. Undoubtedly, androgen metabolism homeostasis in men plays a very important role in maintaining body health physically and psychologically. As we focus on the role of androgen in women suffering with PCOS, that thesis is beyond the scope of our review. But we still briefly summarized it in Figure 5. As shown, excessive androgen in men would lead to a high risk of kidney diseases,^104^, ^106^ prostate cancer^108^ and alopecia,^7^ as well as mental and behaviour problems,^109^ whereas men suffering with androgen deficiency tend to develop into cardiovascular diseases,^76^, ^110^ diabetes^68^ and obesity.^57^

## 5 SUMMARY

Hyperandrogenism is not only an important clinical feature in PCOS but also plays a critical role in the occurrence and development of PCOS. To clarify its origin and effects is of great importance to identify the targeted inhibition on PCOS. We review the physiological role of androgen in women and the pathological role of androgen in PCOS, introduce the involved signals and highlight the role of androgen in PCOS‐related complications. Considering the poor effects of anti‐androgen therapies in clinical settings nowadays, it is crucial to clarify the regulatory mechanisms of androgen in PCOS to establish the best strategies for targeted inhibition.

## CONFLICT OF INTEREST

The authors declare that the research was conducted in the absence of any commercial or financial relationships that could be construed as a potential conflict of interest.

## AUTHOR CONTRIBUTION

**Wenting Ye:** Data curation (supporting); Resources (supporting); Software (supporting). **Tingting Xie:** Data curation (supporting). **Yali Song:** Conceptualization (equal); Data curation (equal); Funding acquisition (equal). **Lili Zhou:** Conceptualization (lead); Data curation (lead); Formal analysis (lead); Funding acquisition (lead); Investigation (lead); Methodology (lead); Project administration (lead); Resources (lead); Software (lead); Supervision (lead); Validation (lead); Visualization (lead); Writing‐original draft (lead); Writing‐review & editing (lead).

## Data Availability

The data that support the findings of this study are available from the corresponding author upon reasonable request.
